# A human-serum-free medium can induce more infectious *P. falciparum* gametocytes than a conventional human-serum-containing medium

**DOI:** 10.1038/s41598-024-73843-5

**Published:** 2024-09-27

**Authors:** Kazutoyo Miura, Bingbing Deng, Ragavan Varadharajan Suresh, Yonas T. Gebremicale, Luwen Zhou, Thao P. Pham, Kyle Roche, Ababacar Diouf, Jonathan F. Lovell, Jean-Philippe Julien, Carole A. Long

**Affiliations:** 1 Laboratory of Malaria and Vector Research, National Institute of Allergy and Infectious Diseases, National Institutes of Health, Rockville, MD 20852 USA; 2 Department of Biomedical Engineering, University at Buffalo, State University of New York, Buffalo, NY 14260 USA; 3 Program in Molecular Medicine, The Hospital for Sick Children Research Institute, Toronto, ON M5G 0A4 Canada; 4 Departments of Biochemistry and Immunology, University of Toronto, Toronto, ON M5S 1A8 Canada; 5 Laboratory of Malaria and Vector Research, National Institute of Allergy and Infectious Diseases, National Institutes of Health, 12735 Twinbrook Parkway, Rockville, MD 20852 USA; 6 Present Address: Academic Programs, the Foundation for Advanced Education in the Sciences, Bethesda, MD 20892 USA

**Keywords:** *Plasmodium Falciparum*: malaria, Gametocyte, Gametocyte culture, Serum free, Standard membrane-feeding assay, SMFA, Transmission-blocking, Parasitology, Vaccines

## Abstract

Malaria remains a global health problem, and the standard membrane feeding assay (SMFA) is a key functional assay for development of new interventions to stop malaria transmission from human to mosquito. For SMFA, media with ~ 10% of human serum has been used for infectious gametocyte cultures, however, there are multiple challenges to obtain a suitable human serum. Here we show a human-serum-free culture medium (HSF), which was a mixture of two stem cell culture media and AlbuMAX, supported infectious gametocyte growth. Moreover, the HSF-induced gametocytes elicited significantly higher numbers of oocysts compared to gametocytes cultured with conventional human serum medium (Conv). While some caution is required when comparing percent transmission reducing activity data generated from HSF-SMFA and Conv-SMFA, the HSF method can facilitate the establishment of gametocyte cultures or SMFA by bypassing the need for human serum. Thus, this study will support future development of *P. falciparum* transmission-blocking interventions.

**Supplementary Information:**

The online version contains supplementary material available at 10.1038/s41598-024-73843-5.

## Introduction

Malaria remains one of the biggest health problems globally. The World Health Organization (WHO) estimated 249 million malaria cases and 608,000 malaria-related deaths in 2022 ^1^, the majority of them caused by one species of malaria, *Plasmodium falciparum*. Although a significant reduction in malaria morbidity and mortality had been seen from 2000 to 2015, progress in malaria control has stalled subsequently^1^. Therefore, to reach the goal of global technical strategy (GTS) defined by the WHO (i.e., to reduce malaria case incidence and mortality rate by at least 90% by 2030 compared to 2015), new interventions are likely to be required.

Transmission-blocking vaccines (TBVs) target the early stages of parasite development in the mosquito, and the transmission stage is a biological bottleneck in the malaria life cycle^2^. Not only TBV, but also monoclonal antibodies^3^ and antimalarial drugs^4–6^, which block human to mosquito transmission, have been under investigation. For such research, a standard membrane feeding assay (SMFA) has been utilized for functional evaluation of transmission blocking interventions, while direct membrane feeding assay (DMFA) and direct skin feed (DSF) have also be used. In SMFA, cultured gametocyte parasites are fed with a test antibody or drug to mosquitoes, and then the reduction in prevalence (transmission blocking activity, TBA) or intensity (transmission reducing activity, TRA) of oocysts compared to a control group is assessed as the efficacy of the test antibody/drug. To perform SMFA, culturing infectious gametocytes is a crucial step, and it has been shown that an error range (e.g., 95% confidence interval, 95%CI) in observed percent TRA (%TRA) value becomes larger when the average number of oocysts in the control group is lower^7,8^. Therefore, to obtain meaningful results from SMFA, it is critical to have a higher number of oocysts in the control groups. Protocols for culturing infectious *P. falciparum* gametocytes were established in the early 1980’s^9,10^, and similar protocols have been used since^11,12^. All such protocols use ~ 10% human serum for the culture medium. However, obtaining human serum for gametocyte cultures can be a major obstacle to conduct research for transmission-blocking interventions in certain areas/countries, and not all human sera support infectious gametocyte growth^12^. For asexual parasite cultures, AlbuMAX, a lipid-rich bovine serum albumin, has been used widely to replace human serum. On the other hand, based on a recent meta-analysis where 19 *P. falciparum* gametocyte culture protocols published from 2000 to 2022 were examined, there was no report using a human-serum-free culture medium to grow infectious *P. falciparum* gametocytes^13^. Of the 19 publications, only three protocols used human-serum-free medium and none of these three studies reported the infectivity of resulting gametocytes. On the other hand, in seven studies where infectivity of gametocytes was confirmed by SMFA, all protocols used 10% human serum (with or without AlbuMAX). In this study, we established a human-serum-free *P. falciparum* gametocyte culture medium.

## Results and discussion

### Development of human-serum-free media for gametocyte and asexual cultures

As published previously^14^, we have used a conventional complete medium (Conv), which includes 10% human serum, both for asexual and gametocyte cultures to conduct SMFA. To develop a human-serum-free gametocyte culture medium which could make infectious gametocytes, several stem cell culture media were first tested to replace 10% human serum. In the preliminary study, a medium called “SR3A medium” showed some promising results. The SR3A medium was a mixture of 2% (v/v) 50x Serum Replacement 3 (i.e., 1x concentration in the final medium) and 0.5% (w/v) AlbuMAX in incomplete culture medium (the incomplete culture medium was Conv minus 10% human serum). The details of individual media are described in Methods section. Compared to gametocytes cultured with Conv, those with SR3A showed significantly fewer oocysts when the cultures were fed to the same batches of mosquitoes (medium of ~ 85% reduction in oocyst density based on eight independent experiments, *p* = 0.008 by a Wilcoxon matched-pairs signed rank test; Supplementary Fig. 1a). Since fewer oocysts in the control is a serious problem in SMFA to obtain reliable results^7,8^, further improvement was pursued.

**Fig. 1:**
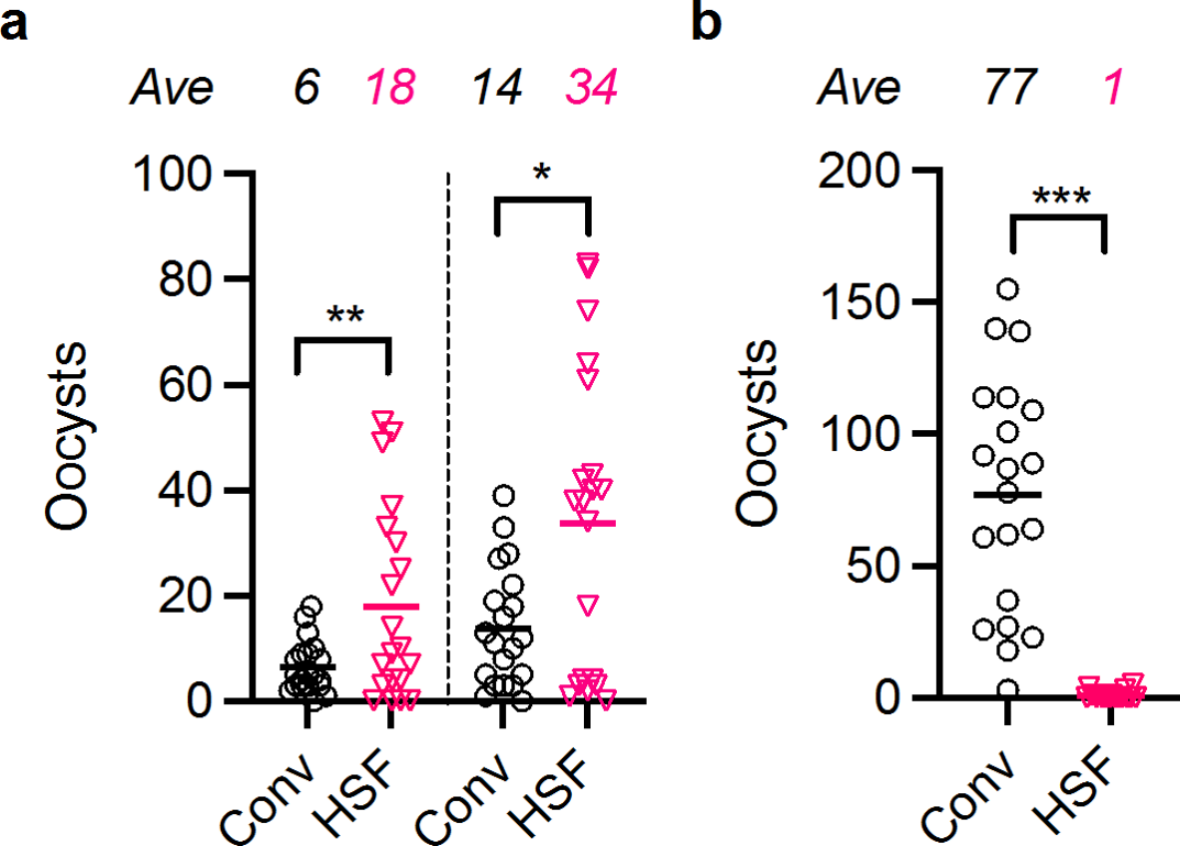
Gametocytes cultured with human-serum-free medium (HSF) or conventional 10% human serum medium (Conv). (**a**) HSF and Conv medium conditions for gametocyte cultures were compared using *Plasmodium falciparum* NF54 parasites. The asexual culture which was maintained using Conv was split into two conditions when gametocyte cultures were initiated, then the two groups of parasites were maintained either with HSF or Conv for ~ 17 days. The two cultures were fed separately to two groups of the same batch of *Anopheles stephensi* mosquitoes. Eight days after the feed, *n* = 20 mosquitoes were dissected per group to enumerate oocysts in each mosquito. Oocyst number in each mosquito (dots), average numbers (bars and numbers above the graph), and a statistical difference calculated by a zero-inflated negative binomial (ZINB) model^8^ are shown. *, *p* < 0.05; **, *p* < 0.01; ***, *p* < 0.001. The results from two independent experiments are presented. (**b**) A similar experiment was conducted as done in **a**. The only difference was that in **a**, SR3A and MSC media were kept separately at 4 °C, and the two media were freshly mixed just prior to daily medium change, while in **b**, SR3A and MSC media were mixed on day 0 of gametocyte culture and the mixture was maintained at 4 °C until used for daily medium change.

In a separate study, we had shown that gametocytes co-cultured with mesenchymal stem cells (MSC) elicited significantly higher numbers of oocysts compared to gametocytes without co-culture^15^. In that study, we used a 1:1 mixture of MSC medium and Conv to maintain both MSC and gametocytes, and there was an insignificant difference in oocyst density between the 1:1 mixture and (100%) Conv culture conditions in two independent experiments^15^. The results indicated the MSC medium was not toxic for gametocytes at least when used at 50% (v/v), while some cell culture media were toxic at the 50% (v/v) concentration^15^. Based on these prior studies, we tested a 1:1 mixture of SR3A medium and MSC medium, absent any human serum, for gametocyte culture and fed the parasites to mosquitoes. In two independent experiments, gametocytes with the 1:1 mixture medium, called “HSF” (human-serum-free culture medium), induced significantly higher oocysts than those with Conv (median of ~ 2.5-fold increase in mean oocysts, *p* < 0.02 by a zero-inflated negative binomial (ZINB) model^8^ for both experiments, Fig. 1a). In the two experiments, SR3A and MSC media were kept separately at 4 °C (up to 3 weeks) and a sufficient volume of mixture was made daily just prior to medium change. Interestingly, when a mixture of the same two media was made on the day of initiating gametocyte culture, and the mixture was maintained at 4 °C (also up to ~ 3 weeks), the long-term-mixed medium did not support infectious gametocyte growth (Fig. 1b). The results suggest that some element(s) in the HSF may not be stable when two media are mixed, a toxic factor(s) may be generated in the long-term-mixed medium, or a combination of both. For all the following experiments, SR3A and MSC media were kept separately and freshly mixed just prior to use. This study did not investigate the mechanism responsible for the difference, but to further improve HSF, a comparison between fresh-mixed and long-term-mixed HSF might be helpful.

The above experiments used HSF for gametocyte cultures, while Conv was still used for upstream asexual culture. To eliminate human serum from the entire culture steps, HSF was next tested for asexual culture. When HSF was used to maintain asexual parasites which had been cultured in Conv for > 2 decades, the parasites grew normally (i.e., ~ 10-fold proliferation every 48 h) from the first proliferation cycle after switching from Conv to HSF. After two weeks of asexual culture in HSF, gametocyte culture was initiated using the same HSF. Contrary to our prediction, the HSF-maintained parasites (both for asexual and gametocyte cultures) showed significantly fewer oocysts than parasites maintained with Conv (both for asexual and gametocyte cultures); with average oocysts of 3 vs. 57, respectively (*p* < 0.001 by the ZINB model; Supplementary Fig. 1b). Therefore, a 0.5% (w/v) AlbuMAX medium (without human serum), which has been widely used for asexual cultures, was evaluated next (refer to Methods section for details of 0.5% (w/v) AlbuMAX medium). Initially, parasites had not grown well in the AlbuMAX medium (~ 2-3-fold proliferation every 48 h), but after ~ 2 weeks of asexual culture, the proliferation rate returned to ~ 10-fold, and gametocyte culture was initiated using HSF. The 0.5% (w/v) AlbuMAX (asexual culture) followed by HSF (gametocyte culture) parasites elicited ~ 2-fold higher average oocysts (average of 42) compared to Conv-maintained parasites (both for asexual and gametocyte cultures, average of 17, *p* = 0.032 by the ZINB model).

## Effect of HSF on the number of oocysts and %TRA

**Fig. 2:**
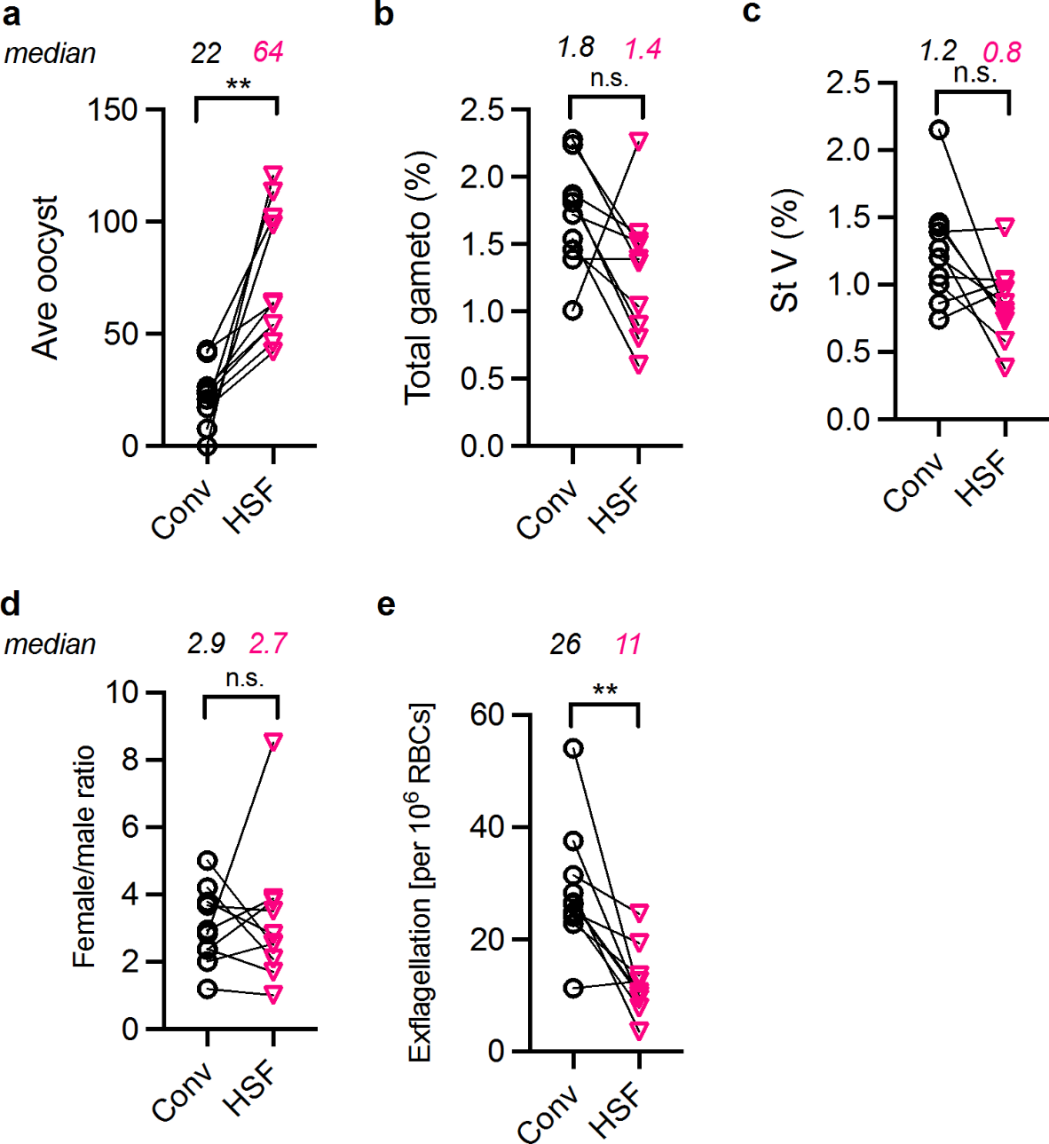
Difference between HSF and Conv. (**a**) Average number of oocysts in each experiment (dots) and median number of average oocysts from 10 independent experiments (numbers above the graph) are shown. Conv and HSF data tested in the same experiment are connected by the line. The total gametocytemia (**b**), stage V gametocytemia (**c**), female and male stage V gametocyte ratio (**d**), and number of exflagellation centers (**e**) in each group in each experiment on the day of feed are shown with median values from 10 experiments. In each panel, a statistical difference between two groups was calculated by a Wilcoxon matched-pairs signed rank test. n.s., not significant; **, *p* < 0.01.

Based on the above results, we generated frozen stocks of AlbuMAX-adapted NF54 parasites, and the following experiments were conducted using the AlbuMAX-adapted parasites. After thawing, the parasites were maintained with the 0.5% (w/v) AlbuMAX medium for asexual culture (up to two months after thawing), and HSF for gametocyte culture. Hereafter, it is called “HSF” group. As a control, the original NF54 parasites (non-AlbuMAX-adapted) which were maintained using Conv both for asexual and gametocyte cultures (called “Conv” group) were included in each experiment. In each experiment, the same batch of RBC and mosquitoes were utilized for both groups. A total of 10 independent experiments (including the one mentioned above) were conducted, and in all 10 experiments, the HSF group elicited higher oocysts than the Conv group (median of 2.4-fold), and the difference was statistically significant (*p* = 0.002 by a Wilcoxon matched-pairs signed rank test; Fig. 2a). In each SMFA, both HSF and Conv gametocyte cultures were diluted to the same stage V gametocytemia (ranging from 0.15 to 0.25% in different experiments; Supplementary Table 1) and fed to mosquitoes. Therefore, the difference in the oocyst numbers was not due to differences in quantity of mature gametocytes in the feeders. Instead, there appears to be a difference in the quality of induced mature gametocytes between the two groups. Despite the higher numbers of oocysts, exflagellation numbers in the HSF group on the day of feed were significantly lower than those in the Conv group (*p* = 0.004 by a Wilcoxon matched-pairs signed rank test; Fig. 2e). The results suggest that mature female gametocytes in the HSF group might be superior to those in the Conv group. However, in contrast to the exflagellation test for mature male gametocytes, there is no method to assess the biological activity of mature female gametocytes. Thus, further studies will be required to uncover the mechanism of higher oocysts observed in the HSF group. While some trends existed, differences in total gametocytemia (Fig. 2b) and stage V gametocytemia (Fig. 2c) were insignificant, and no obvious differences were observed for female/male ratios (Fig. 2d), proportion of gametocyte stages, and shapes of gametocytes (Supplementary Fig. 2) between the two groups. The original data for each experiment are seen in Supplementary Table 1.

**Fig. 3:**
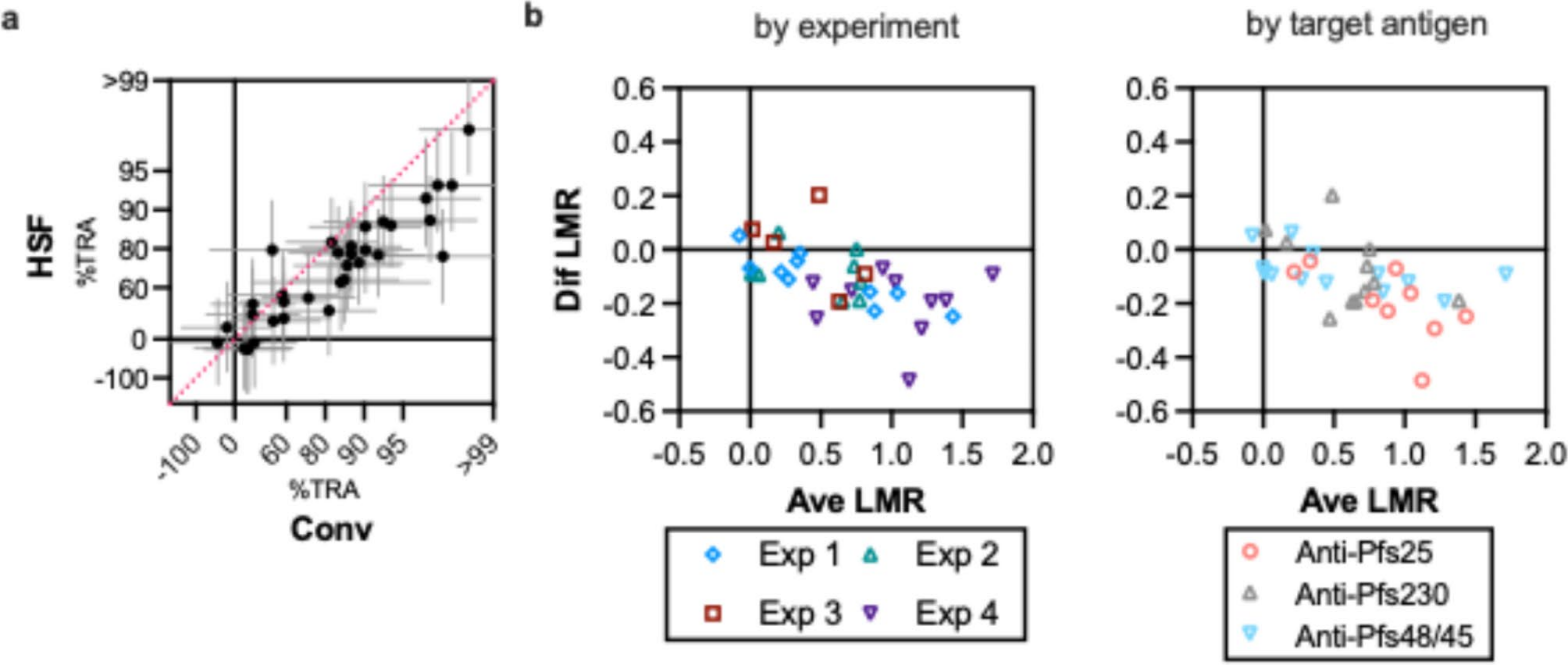
Difference in inhibitory activity between HSF and Conv. (**a**) 16 different antibodies were tested by SMFA using HSF and Conv gametocyte cultures side-by-side. Each antibody was tested at one to three concentrations in one or two independent assays, and a total of 34 paired results were generated. Each dot represents one paired result (error bars show 95% confidence intervals), and the red dotted line shows a y = x line. While the figure is drawn using a log of mean oocyst ratio (LMR; Log_10_ (mean oocyst in control/mean oocyst in test)) scale, corresponding %TRA levels are shown for convenience. (**b**) bland Altman plots are shown. For each paired data set, an average LMR of the HSF and Conv conditions (x-axis), and a difference between the average and HSF (Dif LMR; y-axis) was calculated. A negative Dif LMR means that LMR in HSF was smaller than that in Conv. The same data are grouped by experiment (left) or by target antigen (right).

Finally, the impact of HSF culture on %TRA was evaluated using 16 different antibodies in four independent experiments, and each antibody was tested at one to three concentrations in one or two independent assays (a total of 34 paired %TRA results were generated). The antibodies included two mouse anti-Pfs25 polyclonal antibodies (pAb)^16,17^, 4B7 (anti-Pfs25 monoclonal antibody (mAb))^14^, six mouse anti-Pfs230 pAb^16–18^, six mouse anti-Pfs48/45 pAb^19^ and TB31F (humanized anti-Pfs48/45 mAb)^3^. The results of SMFA (%TRA value and the 95%CI) for individual antibodies in individual experiments are seen in Supplementary Table 2. There was a strong correlation in inhibitory activities between Conv and HSF (the Spearman coefficient was 0.88 and *p* < 0.0001 by a Spearman rank test; Fig. 3a), and 95%CI of %TRA estimates between the two conditions overlapped each other in 33 out of 34 paired data sets (one exception was one mouse anti-Pfs25 pAb which showed 98%TRA in Conv and 77%TRA in HSF; Supplementary Table 2). However, when all data were combined, the HSF condition showed a significantly lower inhibitory activity (*p* < 0.001 by a Wilcoxon matched-pairs signed rank test), regardless of experiments or target antigens (Fig. 3b). The results suggest while inhibitory activities among different samples can be compared reasonably within the same culture condition, SMFA with HSF would slightly underestimate an inhibitory activity of a test sample compared to SMFA with Conv. However, the difference in %TRA estimates between the two conditions was relatively small compared to the intrinsic uncertainty of SMFA, i.e., 97% (33/34) paired data demonstrated overlapped 95%CI of %TRA estimates (the 95%CI was calculated by a SMFA-specific ZINB model, which was generated using data from > 10,000 mosquitoes tested in 110 feeds^8^). Thus, a large study is likely to be required to answer whether any modification(s) in culture or feeding procedures could diminish the small difference in %TRA between the two medium conditions.

There are several limitations in this study. First, Serum Replacement 3 and MSC basal medium (the main part of MSC medium) are proprietary products, thus the contents cannot be specified. However, this is also true for human serum. Importantly, the new HSF medium does not require a screening step to select suitable batch(es) of products for gametocyte culture, while such screening is necessary for human serum^12^. Second, while the new culture method does not require human serum during asexual and gametocyte cultures, all SMFA conducted in this study still used human serum for the feeding experiments as a source of human complement. It is well-documented that a majority of anti-Pfs230 antibodies show complement-dependent inhibition in SMFA, and anti-Pfs230 antibodies tested in this study also demonstrated complement-dependent inhibition with HSF gametocytes as predicted (Supplementary Fig. 3). Compared to the amount of serum required for asexual and gametocyte culture, the volume of serum used for a feed is much smaller (typically we use 4 mL of human serum for ~ 17 days of gametocyte culture per test sample, but only 0.1 mL of serum for the feed). However, if one would want to conduct completely human-serum-free SMFA (i.e., including the feeding process), especially for complement-dependent antibodies, further modification would be required. Finally, this study was conducted using a single strain of NF54 parasites. Future studies are anticipated to determine whether the new HSF-based protocol works for other strains of *P. falciparum* parasites (and/or NF54 parasites from different laboratories).

The newly developed human-serum-free gametocyte culture medium (HSF) can provide more infectious *P. falciparum* gametocytes than a conventional 10% human serum culture medium (Conv). While some caution is required when comparing %TRA data generated from HSF-SMFA and Conv-SMFA, the HSF method will help researchers establish gametocyte culture or SMFA without the difficulties associated with obtaining suitable batches of human serum. Furthermore, higher infectivity with HSF gametocytes is likely to increase the accuracy of %TRA estimates of test samples in SMFA^7,8^. Thus, HSF can support biological research with *P. falciparum* gametocytes and future development of transmission-blocking interventions.

## Methods

### Reagents

Incomplete RPMI-1640 culture medium (IncoCM; Catalog number, Cat # CUS-0645), which contained L-Glutamine, 25mM HEPES and 50 mg/L hypoxanthine was purchased from K. D. Medical (Columbia, MD, USA). AlbuMAXII, sodium bicarbonate (7.5% solution) and culture flasks (Falcon 353136) were obtained from Thermo Fisher Scientific (Waltham, MA, USA), and Serum Replacement 3 (Cat#, S2640) was from Sigma-Aldrich (Rockville, MD, USA). Mesenchymal Stem Cell Basal Medium for Adipose, Umbilical and Bone Marrow-derived MSCs (Cat# PCS-500-030) and Mesenchymal Stem Cell Growth Kit for Bone Marrow-derived MSCs (Cat# PCS-041) were purchased from ATCC (Gaithersburg, MD, USA). For malaria cultures, deidentified human O + type sera and red blood cells (RBC) were obtained from GRIFOLS BioSupplies (Memphis, TN, USA).

## Asexual and gametocyte cultures using conventional 10% human serum complete medium

For all experiments, *P. falciparum* NF54 strain parasites were used in this study, and the culture method using conventional 10% human serum complete medium (Conv) has been published previously^14^. The human sera for Conv culture were obtained from malaria naïve US adults, and the sera were screened for suitability for gametocyte cultures before use. All cultures, including human-serum-free medium cultures, were conducted in a static condition. For both asexual and gametocyte cultures, the same culture medium was utilized: IncoCM with 2.5 g/L of sodium bicarbonate and 10% human serum (no antibiotics). The asexual culture was maintained at 2% hematocrit (Ht) without synchronization up to two months after thawing from a frozen parasite stock, and diluted every 2–3 days to keep the parasitemia lower than 10%. The gametocyte culture was initiated using the asynchronized asexual cultures with 0.5–0.7% parasitemia at 5% Ht, and with 0.2–0.4% parasitemia at 2% Ht in the volume of 30 mL. The initiation of gametocyte cultures was performed on two consecutive days for each experiment (a total of 4 culture flasks per experiment). All cultures were maintained in an atmosphere of 5% O_2_, 5% CO_2_ and 90% N_2_ for 16–18 days with daily medium change. There was no addition of fresh uninfected RBC, except for the following procedures. For the 5% Ht culture flasks, half of the culture (i.e., 15 mL of culture) was discarded on day 2 of gametocyte culture, and 750 µL of fresh uninfected RBC and 15 mL of fresh medium were added (i.e., the final volume of 30 mL with 5% Ht). For the 2% Ht culture flasks, 900 µL of fresh RBC was added on day 3 to make 5% Ht cultures (again, the final volume of 30 mL with 5% Ht). On day ~ 8 of gametocyte cultures, total gametocytemia (stage III dominant; Supplementary Fig. 2a) in each flask was evaluated microscopically, and if one or two flask of cultures showed much lower gametocytemia than the others (usually if total gametocytemia was less than 0.4 to 0.6%), the cultures were terminated. The remaining cultures were maintained until day 16–18 for SMFA.

### Asexual and gametocyte cultures using human-serum-free medium

Asexual *P. falciparum* NF54 parasites, which had been maintained in Conv (with 10% human serum) for > 2 decades at NIAID, were cultured for ~ 2 weeks using an AlbuMAX-based (human-serum-free) asexual culture medium. The asexual culture medium was a mixture of IncoCM with 0.5% (w/v) AlbuMAXII and 0.225% sodium bicarbonate. After ~ 2 weeks of culture, frozen stocks of “AlbuMAX-adapted” NF54 parasites were generated. The protocol to maintain the asexual and gametocyte cultures was identical as above, except for culture media. For asexual cultures, the same AlbuMAXII-based medium was used. For gametocyte cultures, two culture media were prepared separately. One (called “SR3A” medium) was a mixture of 10 mL of Serum Replacement 3, 30 mL of 7.5% sodium bicarbonate, 5 g of AlbuMAXII and 960 mL of IncoCM. The other was MSC medium, which was made following ATCC’s instruction by mixing the MSC basal medium and MSC growth kit (MSC basal medium with 7% FBS, 15 ng/mL of rhIFG-1, 125 pg/mL of rh FGF-b and 2.4 mM L-alanyl-L-glutamine). The tubes/bottles containing the complete MSC medium were covered by aluminum foil until used to minimize exposure to light. Both media were kept at 4 °C separately for up to 3 weeks, and they were mixed at a 1:1 volume ratio just prior to use on each day of medium change. The 1:1 mixture of two media is called human-serum-free culture medium (HSF) in this manuscript.

## SMFA

The detailed methodology of SMFA has been published elsewhere^14^. In each feeding experiment for each medium condition, one to three cultures (out of 4-flask of initiated cultures) were selected based on their stage V gametocytemia and exflagellation activities. A Giemsa slide was made for each culture, and the numbers of stage III, IV, and male or female stage V gametocytes were determined by morphology by examining 2,500-5,000 RBCs. For an exflagellation test, 0.2–0.3 mL of culture was transferred to a tube, spun down, and aspirated the supernatant to make a 50% Ht suspension. An equal volume of heat-inactivated normal human serum was added to the suspension (became 25% Ht), and incubated for 20 min at 19 °C. The mixture was transferred to a cellometer cell counting slide, and the number of exflagellation centers was determined. Based on the number and 25% Ht, the exflagellation centers per 10^6^ RBCs were calculated. If more than one culture was selected per medium condition, then pooled gametocyte culture was made. The selected (pooled) culture was centrifuged at 2,000xg for 10 min, the medium was replaced with normal human serum (O + type), and fresh RBC was added to make a gametocyte mixture with 0.2 +/- 0.05% stage V gametocytemia at 50% Ht. For each experiment, total gametocytemia, stage V gametocytemia, female and male stage V gametocyte ratio, and exflagellation numbers of each culture on the day of feed, and final stage V gametocytemia fed to mosquitoes are seen in Supplementary Table 1. For feeding experiment, 60 µl of normal human serum or a test sample (a defined concentration of test antibody diluted in 1x phosphate buffered saline) was mixed with 200 µl of the gametocyte mixture (50% Ht with human serum as described above), and the final mixture was immediately fed to ~ 50 of 3–6 days old female *Anopheles stephensi* (Nijmegen strain) mosquitoes through a membrane feeding apparatus. The mosquitoes were kept for 8 days and dissected (*n* = 20 per group) to enumerate the oocysts in the midgut. Only midguts from mosquitoes with any mosquito eggs at the time of dissection were analyzed.

### Statistical analysis

Percent inhibition in mean oocyst intensity (%TRA) was calculated as: 100 x {1 - (mean number of oocysts in the test) / (mean number of oocysts in the control)}. A log of mean oocyst ratio (LMR) was calculated as: Log10 (mean oocyst in the control/mean oocyst in the test). Therefore, LMR = Log_10_ {100/(100-%TRA)}.

Statistical difference in oocyst numbers between two groups was assessed using a zero inflated negative binomial (ZINB) model described before^8^. To compare two paired data sets from two different groups in multiple independent experiments, a Wilcoxon matched pairs signed rank test was used. For correlation analyses, a Spearman rank test was utilized. All analyses were two-tailed tests conducted either by GraphPad Prism version 9.3 (GraphPad Software) or R version 4.3.3 (The R Foundation for Statistical Computing), and p ˂ 0.05 was considered as significance.

## Electronic supplementary material

Below is the link to the electronic supplementary material.

Supplementary Material 1

## Data Availability

All original data generated during this study are included in this publication (and its supplemental material).
