# Supplementary material for: A human-serum-free medium can induce more infectious P. falciparum gametocytes than a conventional human-serum-containing medium
Source: Sci Rep. 2024 Sep 27;14:22052. doi: 10.1038/s41598-024-73843-5 (PMC11436888; doi:10.1038/s41598-024-73843-5)
Supplement: Supplementary file 1 — Supplementary Material 1 [file 41598_2024_73843_MOESM1_ESM.pdf]

A human-serum-free medium can induce more infectious *P. falciparum* gametocytes than a conventional human-serum-containing medium.

Kazutoyo Miura, Bingbing Deng, Ragavan Varadharajan Suresh, Yonas T. Gebremicale, Luwen Zhou, Thao P. Pham, Kyle Roche, Ababacar Diouf, Jonathan F. Lovell, Jean-Philippe Julien, Carole A. Long

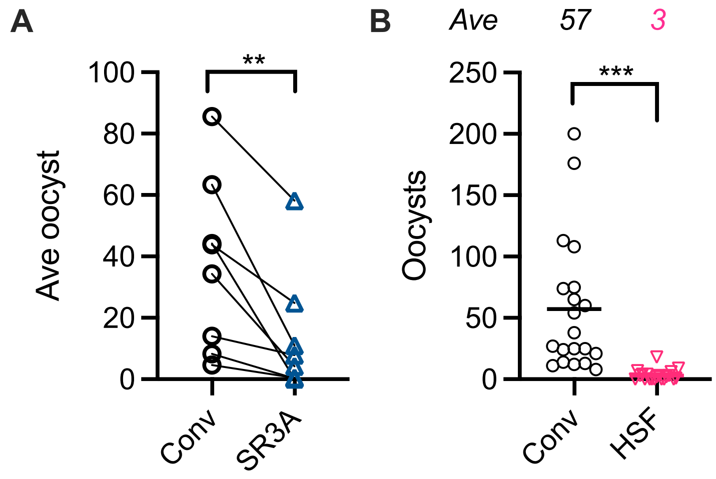

Figure S1. Oocyst data in unoptimized culture conditions. (**A**) In each independent experiment, *P. falciparum* NF54 gametocyte parasites were maintained with either Conv (which included 10% human serum) or SR3A (which included Serum Replacement 3 and AlbuMAX, instead of 10% human serum), and the parasites were fed to two groups (but the same batch) of mosquitoes separately. Eight days after the feed, n=20 mosquitoes were dissected per group, and average oocyst number was calculated. Conv and SR3A data tested in the same experiment are connected by a line, and results of eight independent experiments are shown with p-value calculated by a Wilcoxon matched-pairs signed rank test. **, P<0.01. (**B**) Asexual and gametocyte cultures were conducted using Conv for one group, and HSF was used for asexual and gametocyte cultures for the other group. Oocyst number in each mosquito (n=20 per group), average oocyst number (bar and number above the graph), and a statistical difference calculated by a zero-inflated negative binomial model [1] are shown. ***, P<0.001. A single experiment was conducted.

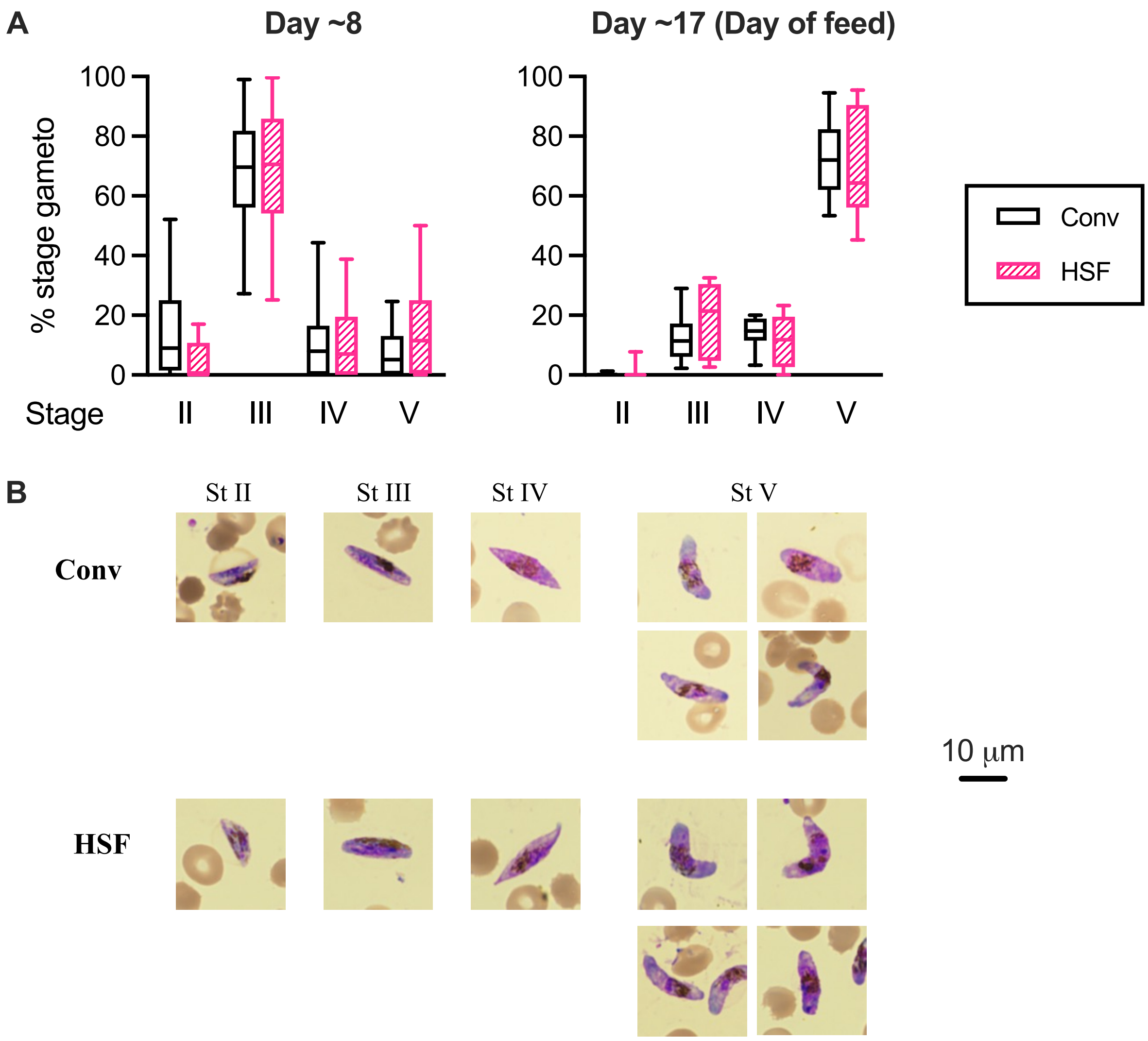

Figure S2. Comparison between Conv and HSF groups. (**A**) A proportion of each stage of gametocytes within total gametocytes was evaluated on days ~8 (middle of gametocyte culture) and ~17 (day of feed) in 10 independent experiments. A box plot (25, 50 and 75 percentile) with error bar (2.5 and 97.5 percentile) are shown representing stages II through V of gametocyte development. (**B**) Representative images of fixed and Giemsa-stained each stage of gametocytes in the two groups.

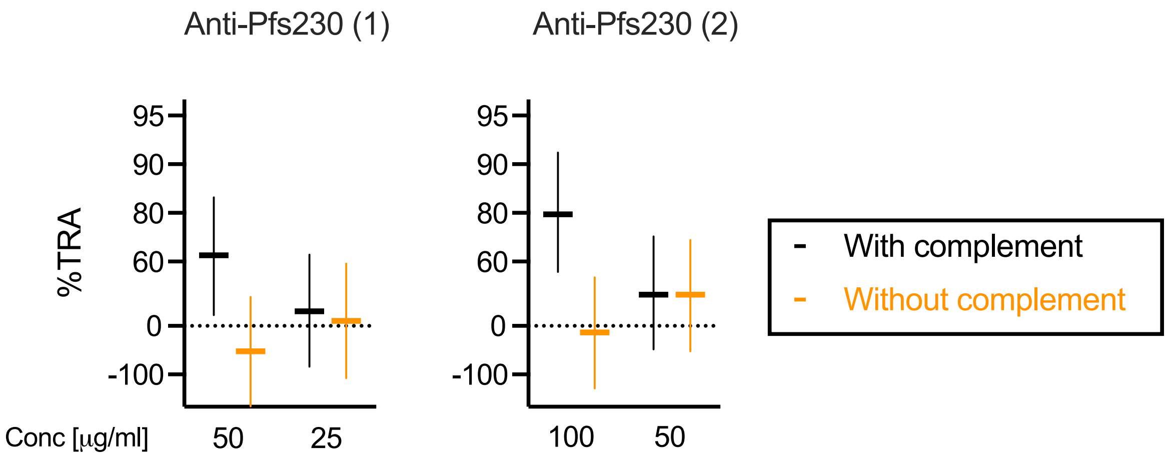

Figure S3. Complement dependent inhibition in SMFA with HSF gametocytes. Two mouse anti-Pfs230 antibodies were tested by SMFA at 50/25 or 100/50 μg/mL concentrations using HSF gametocytes with (non-heat-inactivated human serum) or without (heat-inactivated human serum) human complement. The resulting %TRA in each test condition is shown with the 95%CI.

Reference

1. Miura K, Swihart BJ, Deng B, Zhou L, Pham TP, Diouf A, et al. Transmission-blocking activity is determined by transmission-reducing activity and number of control oocysts in *Plasmodium falciparum* standard membrane-feeding assay. Vaccine. 2016;34(35):4145-51.

Table S1; Original data for Figure 2

Exp # [1]	Conv [2]					HSF [3]					Feed St V [4]
	Total Game [5]	St V [6]	F/M ratio [7]	Exfla [8]	Ave Ooc [9]	Total Game [5]	St V [6]	F/M ratio [7]	Exfla [8]	Ave Ooc [9]	
364-1	2.24	1.39	2.38	24.8	17.1	1.50	1.42	1.69	19.3	41.9	0.18
366-1	1.39	0.74	1.19	26.3	26.6	1.39	0.96	1.00	10.0	54.2	0.17
370-1	1.01	0.86	2.36	11.3	0.1	2.26	1.02	3.80	12.5	120.5	0.25
373-2	1.85	1.46	2.83	28.3	42.9	0.80	0.73	8.50	3.5	63.2	0.20
378-1	1.81	1.20	5.00	31.4	41.7	0.90	0.86	2.50	24.5	101.6	0.18
382	1.54	1.27	3.67	26.5	20.9	0.60	0.38	3.50	10.5	46.2	0.18
387-1	1.72	1.06	4.20	22.8	17.1	1.52	1.03	2.08	13.5	113.3	0.20
388-1	1.87	1.43	2.94	37.5	7.8	1.58	0.77	3.88	9.4	98.5	0.20
389	2.28	2.15	3.78	54.0	24.8	1.36	0.80	2.81	10.5	64	0.18
393-1	1.46	1.00	2.00	24.0	23.4	1.04	0.58	2.54	7.8	54.2	0.20

[1] Experiment number

[2] Conventional culture medium (10% human serum)

[3] Human-serum-free medium

[4] Stage V gametocytemia in a feeder (in each experiment, both Conv and HSF cultures were diluted to the same targeted St V gametocytemia)

[5] Total gametocytemia (%) on the day of feed

[6] Stage V gametocytemia (%) on the day of feed

[7] Female and male stage V gametocoyte ratio on the day of feed

[8] Number of exflagellation center (per 10^6 RBCs) on the day of feed

[9] Average oocysts (n=20 per group)

Table S2; Original data for Figure 3

Exp # [1]	Sample name	IgG conc [ug/ml]	Conv [2]						HSF [3]						95%CI Overlap [4]
			Cont Ave [5]	Test Ave [6]	%TRA	95%CI Lo [7]	95%CI Hi [8]	p-value [9]	Cont Ave [5]	Test Ave [6]	%TRA	95%CI Lo [7]	95%CI Hi [8]	p-value [9]	
Exp 1	Anti-Pfs25 (1)	94	9.6	0.8	92.2	82.8	96.7	0.001	40.7	9.1	77.8	52.7	90.1	0.001	Yes
Exp 1	Anti-Pfs25 (1)	47	9.6	4.8	50.1	-15.1	78.4	0.094	40.7	29.8	26.8	-55.2	67.1	0.391	Yes
Exp 1	Anti-Pfs25 (2)	50	9.6	0.2	97.9	94.7	99.5	0.001	40.7	2.7	93.5	85.6	97.5	0.001	Yes
Exp 1	Anti-Pfs25 (2)	25	9.6	0.6	93.8	85.5	97.6	0.001	40.7	5.4	86.9	72.3	94.2	0.001	Yes
Exp 1	Anti-Pfs25 (2)	13	9.6	4.1	57.9	12.6	81.8	0.023	40.7	20.9	48.8	-12.4	76.3	0.097	Yes
Exp 1	Anti-Pfs48/45 (1)	100	9.6	1.0	90.1	77.8	95.8	0.001	40.7	8.3	79.6	55.9	91.1	0.002	Yes
Exp 1	Anti-Pfs48/45 (1)	50	9.6	4.0	58.4	6.9	82.4	0.020	40.7	28.1	31.1	-48.1	68.9	0.339	Yes
Exp 1	Anti-Pfs48/45 (1)	25	9.6	13.0	-34.5	-208.8	39.8	0.470	40.7	43.2	-6.0	-122.3	51.5	0.896	Yes
Exp 1	Anti-Pfs48/45 (2)	50	9.6	4.2	56.9	-4.3	84.6	0.060	40.7	18.8	54.0	3.4	79.3	0.045	Yes
Exp 1	Anti-Pfs48/45 (2)	25	9.6	8.2	14.8	-100.0	64.9	0.690	40.7	47.8	-17.2	-144.1	46.0	0.686	Yes
Exp 2	Anti-Pfs230 (1)	50	8.0	1.2	85.0	45.5	97.7	0.004	71.7	26.2	63.5	14.5	84.0	0.016	Yes
Exp 2	Anti-Pfs230 (1)	25	8.0	9.2	-14.4	-159.2	49.0	0.718	71.7	58.1	18.9	-78.8	63.8	0.637	Yes
Exp 2	Anti-Pfs230 (2)	100	8.0	4.2	48.1	-47.1	84.2	0.194	71.7	14.6	79.6	53.7	91.5	0.001	Yes
Exp 2	Anti-Pfs230 (2)	50	8.0	5.8	27.5	-70.3	67.0	0.458	71.7	45.9	35.9	-39.3	72.0	0.276	Yes
Exp 2	Anti-Pfs48/45 (3)	3	8.0	1.0	87.5	70.6	95.1	0.001	71.7	13.6	81.1	57.9	91.5	0.002	Yes
Exp3	Anti-Pfs230 (3)	100	22.5	2.8	87.5	73.0	94.8	0.001	28.6	6.3	78.2	52.7	90.5	0.001	Yes
Exp3	Anti-Pfs230 (4)	100	22.5	3.2	85.8	65.9	95.1	0.001	28.6	9.9	65.6	26.2	84.6	0.006	Yes
Exp3	Anti-Pfs230 (5)	100	22.5	3.6	84.0	63.3	93.8	0.001	28.6	6.1	78.9	54.1	90.5	0.001	Yes
Exp3	Anti-Pfs230 (6)	100	22.5	4.0	82.2	60.9	92.3	0.001	28.6	5.1	82.4	62.9	91.9	0.001	Yes
Exp3	Anti-Pfs25 (3)	100	22.5	2.5	89.1	76.2	95.1	0.001	28.6	7.4	74.1	45.9	88.2	0.002	Yes
Exp3	Anti-Pfs48/45 (4)	50	22.5	17.9	20.4	-70.4	63.4	0.543	28.6	34.2	-19.5	-157.5	44.9	0.705	Yes
Exp3	Anti-Pfs48/45 (5)	100	22.5	17.9	20.4	-70.5	63.4	0.578	28.6	33.8	-18.1	-164.1	48.2	0.700	Yes
Exp3	Anti-Pfs48/45 (6)	50	22.5	15.8	29.7	-50.4	67.7	0.351	28.6	30.8	-7.4	-135.2	52.5	0.837	Yes
Exp3	Anti-Pfs48/45 (7)	100	22.5	16.4	27.3	-51.8	65.5	0.380	28.6	15.5	45.9	-13.4	76.1	0.113	Yes
Exp 4	Anti-Pfs230 (1)	50	22.4	4.2	81.3	59.6	92.2	0.001	39.9	24.2	39.3	-31.5	72.7	0.204	Yes
Exp 4	Anti-Pfs230 (2)	200	22.4	0.6	97.3	93.1	99.4	0.001	39.9	2.6	93.6	85.3	97.6	0.001	Yes
Exp 4	Anti-Pfs230 (3)	100	22.4	3.0	86.6	68.1	95.3	0.001	39.9	10.8	72.9	41.5	88.4	0.002	Yes
Exp 4	Anti-Pfs25 (1)	94	22.4	0.7	96.9	93.2	98.7	0.001	39.9	4.8	88.0	74.8	94.5	0.001	Yes
Exp 4	Anti-Pfs25 (2)	25	22.4	2.2	90.2	77.2	96.9	0.001	39.9	5.4	86.5	70.5	93.9	0.001	Yes
Exp 4	Anti-Pfs25 (3)	200	22.4	0.6	97.5	93.8	99.6	0.001	39.9	9.2	76.9	47.4	90.0	0.001	No
Exp 4	Anti-Pfs48/45 (1)	100	22.4	1.6	92.9	81.5	97.8	0.001	39.9	5.0	87.6	73.5	94.5	0.001	Yes
Exp 4	Anti-Pfs48/45 (2)	100	22.4	6.1	73.0	39.1	88.1	0.002	39.9	19.0	52.4	-0.3	78.9	0.051	Yes
Exp 4	Anti-Pfs48/45 (3)	3	22.4	0.4	98.4	96.6	99.4	0.001	39.9	1.0	97.6	94.8	99.1	0.001	Yes
Exp 4	Anti-Pfs48/45 (7)	200	22.4	0.8	96.7	92.2	98.9	0.001	39.9	3.3	91.9	81.1	97.2	0.001	Yes

[1] Experiment number
[2] Conventional culture medium (10% human serum)
[3] Human-serum-free medium
[4] Whether 95%CI of %TRA between the two culture conditions overlapped or not
[5] Average oocysts in the control of the feed
[6] Average oocysts in the test
[7] Lower end of 95%CI in %TRA estimate
[8] Higher end of 95%CI in %TRA estimate
[9] Whether observed %TRA was significantly different from no inhibition (zero %TRA)
